# Analytical techniques for characterization of raw materials in cell culture media

**DOI:** 10.1186/1753-6561-5-S8-P5

**Published:** 2011-11-22

**Authors:** Chandana Sharma, Barry Drew, Kevin Head, Rani Pusuluri, Matthew V Caple

**Affiliations:** 1 Cell Sciences and Development, SAFC, 13804 W 107th St, Lenexa, KS 66215, USA

## Abstract

Raw materials are a critical part of any cell culture medium; therefore, it is of utmost importance to understand and characterize them for high-quality product. The raw material characterization (RMC) program at SAFC focuses on individual screening of raw materials both analytically and biologically. The goal of the program is to develop the best-in-class knowledge base of the raw materials used in SAFC’s media formulations and their impact on performance of products.

## Background

A prioritized list of 100 “high-risk” raw materials was developed based on a risk assessment performed within SAFC. This poster will focus on the analytical screening of certain “high-risk” raw materials within the prioritized list to identify any variability and critical contaminants present. In order to achieve this, orthogonal methods were used that include ultra-high performance liquid chromatography-mass spectrometry (U-HPLC/MS) for non-volatile polar components and gas chromatography-mass spectrometry (GC/MS) for volatile non-polar materials. Inductively coupled plasma-optical emission spectrometry (ICP-OES) was also used to identify any trace metal contamination present. In addition, the solubility of the raw materials is also tested to identify any variability within a vendor or between different vendors.

## Results

**Figure 1:**
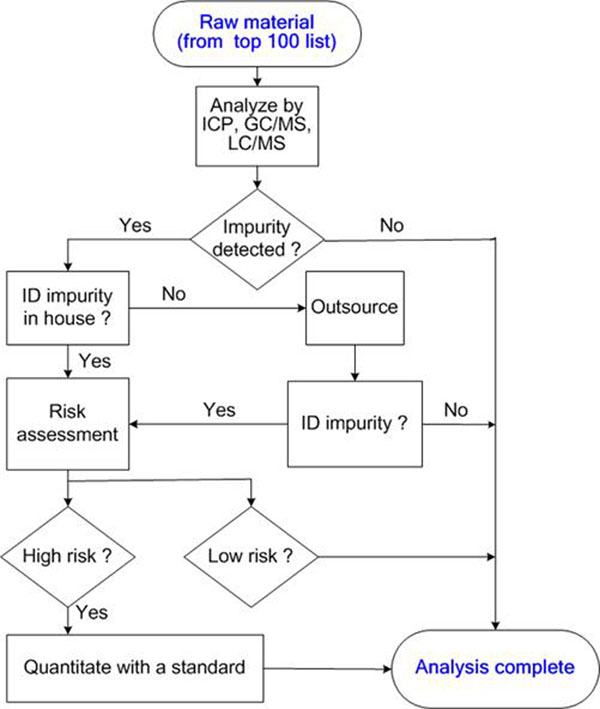
Analytical RMC process flow

Figure 1 shows the process flow followed within the analytical RMC initiative. It describes how each raw material is screened and the strategy for analyzing any possible contaminant.

**Table 1:** Summary of solubility findings on multiple lots of three amino acids.

		Solubility in 100 ml of a neutral buffer		
**Vendor**	**Lot No.**	**Lysine.HCl (g)**	**Cystine.2HCl (mg)**	**Tyrosine.2Na (mg)**

A	1	5.0	12.4	175.1
	2	12.5	35.0	185.8
	3	10.0	35.2	185.5

B	1	15.0	30.3	185.8
	2	12.5	50.8	195.2
	3	12.0	48.2	200.7

C	1	60.0	32.0	N/A
	2	60.0	35.2	N/A
	3	60.0	32.8	N/A

D	1	60.0	N/A	N/A
	2	60.0	N/A	N/A

## Conclusions

The orthogonal methods cited to characterize raw materials have proven to be robust and reliable for the intended purpose. The solubility experiment described for amino acids illustrates a significant difference in solubility limits of amino acids and establishes intra- and inter-vendor variability. This program has helped SAFC get better insight into their suppliers’ manufacturing processes. This is a long term initiative within the organization and the most important goal through the program is to develop a better understanding of raw materials to deliver superior products of the finest quality.

